# Effectiveness of Kinematic Alignment-Total Knee Arthroplasty in Treating Preoperative Varus and Valgus Deformities in Patients With Knee Osteoarthritis

**DOI:** 10.7759/cureus.53230

**Published:** 2024-01-30

**Authors:** Kyle R Davis, Varun Soti

**Affiliations:** 1 Orthopedic Surgery, Lake Erie College of Osteopathic Medicine, Elmira, USA; 2 Pharmacology and Therapeutics, Lake Erie College of Osteopathic Medicine, Elmira, USA

**Keywords:** valgus deformity, varus deformity, patient reported outcomes measurement, implant survival, kinematic alignment, mechanical alignment, total knee arthroplasty (tka), knee osteoarthritis (koa)

## Abstract

Knee osteoarthritis (OA) is surgically treated with total knee arthroplasty (TKA). Traditionally, TKA has been performed using a mechanical alignment (MA) philosophy. However, due to significant patient dissatisfaction with surgical outcomes, an alternate alignment approach, kinematic alignment (KA), has gained popularity. KA-TKAs have improved functional patient outcomes by restoring the patient's native joint line orientation and minimizing soft tissue releases compared to neutral alignment in MA-TKAs. This review explores the postoperative effectiveness of utilizing KA-TKA to treat knee OA in patients with preoperative varus and valgus deformities. A comprehensive literature search was conducted on PubMed and Biomed Central databases, following the Preferred Reporting Items for Systematic Reviews and Meta-Analyses (PRISMA) guidelines. The literature search focused on studies analyzing the postoperative TKA outcomes in knee OA patients with preoperative varus or valgus deformities whose surgeries followed a KA philosophy and those comparing KA-TKA with MA-TKA. The available clinical evidence indicates that KA-TKA can be a viable treatment option for individuals with knee OA. The alignment of phenotypes has little clinical significance concerning functional outcomes and implant survival rates following KA-TKA. Furthermore, surgery outcomes in patients with preoperative deformities who underwent KA-TKA were similar to those who underwent MA-TKA. KA-TKA produced significantly better functional outcomes than MA-TKA in certain aspects. However, studies with larger sample sizes and more extended follow-up periods that directly compare KA-TKA with MA-TKA in treating knee OA patients are needed to fully demonstrate the efficacy of each technique. Furthermore, further research into the effects of KA-TKA on implant survival rates will provide a better understanding of the benefits of this technique and ultimately lead to improved patient outcomes.

## Introduction and background

Knee osteoarthritis (OA) stands as one of the most prevalent degenerative joint diseases, imposing a significant burden on both patients and the healthcare system as a whole [1]. The incidence of knee OA in the United States has steadily increased in recent decades, which is expected to continue [2]. Total knee arthroplasty (TKA) is the most effective treatment for severe knee OA, significantly alleviating pain and improving patients’ quality of life. Projections suggest a 673% increase in demand for TKA by 2030 [3].

Since its inception in 1985, the mechanical alignment (MA) philosophy, aiming to align the limb with a neutral mechanical axis [4], has gained popularity in TKA. This alignment is crucial for optimizing patients’ postoperative recovery [5]. Neutral MA is believed to equalize the load distribution on the implant, leading to reduced wear and increased implant longevity [6].

MA-TKA has undergone significant advancements in surgical techniques and implant design, resulting in improved alignment and implant survivorship accuracy. However, studies indicate that up to 25% of patients remain dissatisfied with the procedure’s outcomes despite improving the techniques and designs [7-9]. This observation is attributed to the fact that mechanically aligned TKA compromises native knee kinematics, which impacts the surgery’s clinical and functional outcomes [10].

Notably, the natural alignment of both arthritic and non-arthritic knees varies significantly. Research indicates that only 15% of arthritic and non-arthritic knees have a neutral MA. In comparison, 32% of non-arthritic males and 17% of females have a natural varus joint line angle greater than 3° [11-12]. Despite this variation, every MA-TKA aims to achieve neutral alignment regardless of the patient’s native alignment. The fixed alignment philosophy of MA-TKA does not account for the preoperative alignment variations, which may explain the significant proportion of patient dissatisfaction after TKA [13].

In 2006, researchers proposed a new alignment philosophy for TKA, kinematic alignment (KA). The primary goal of KA-TKA is to restore the natural tibiofemoral articular surface and align the tibial and femoral component axes with the three kinematic axes of the normal knee instead of generating a neutral mechanical angle [14]. Proponents of KA-TKA suggest that this approach minimizes flexion and extension gap imbalances, removes the need for soft tissue release, relieves postoperative pain, and improves function and patient satisfaction. By preserving a patient’s alignment, KA-TKA may help improve functional and clinical outcomes compared to the MA-TKA philosophy [15-16].

Using KA-TKA as an alternative to traditional MA-TKA is a subject of ongoing research. While this strategy has shown promise in postoperative patient-reported outcomes, some concerns have been raised regarding the deviation from the conventional neutral MA of the tibial component. Some researchers warned that such deviation could result in early implant failure [17]. Additionally, a few expressed concern for the increased risk of polyethylene wear, leading to reduced prosthesis survival [18]. They also noted increased stress at the patellofemoral joint, which could cause patellofemoral joint instability [19].

A group of researchers demonstrated increased varus alignment of the tibial component associated with increased implant migration and condylar liftoff. They concluded that the tibial component should be neutrally aligned to the mechanical axis [20]. Although KA-TKA has shown similar, if not improved, postoperative patient-reported outcomes, many surgeons remain hesitant to utilize this approach for every patient, regardless of the patient’s preoperative limb alignment [21-23].

This systematic review aims to evaluate the efficacy of KA-TKA for treating knee OA in patients with preoperative varus and valgus deformities. It examines clinical evidence relating to patient-reported outcome measures (PROMs) and survivorship of KA-TKA across varying native knee phenotypes. This will provide substantial information that may aid surgeons and healthcare teams decide when to choose the appropriate alignment philosophy for each patient when performing a TKA.

## Review

Data sources and study selection 

**Figure 1:**
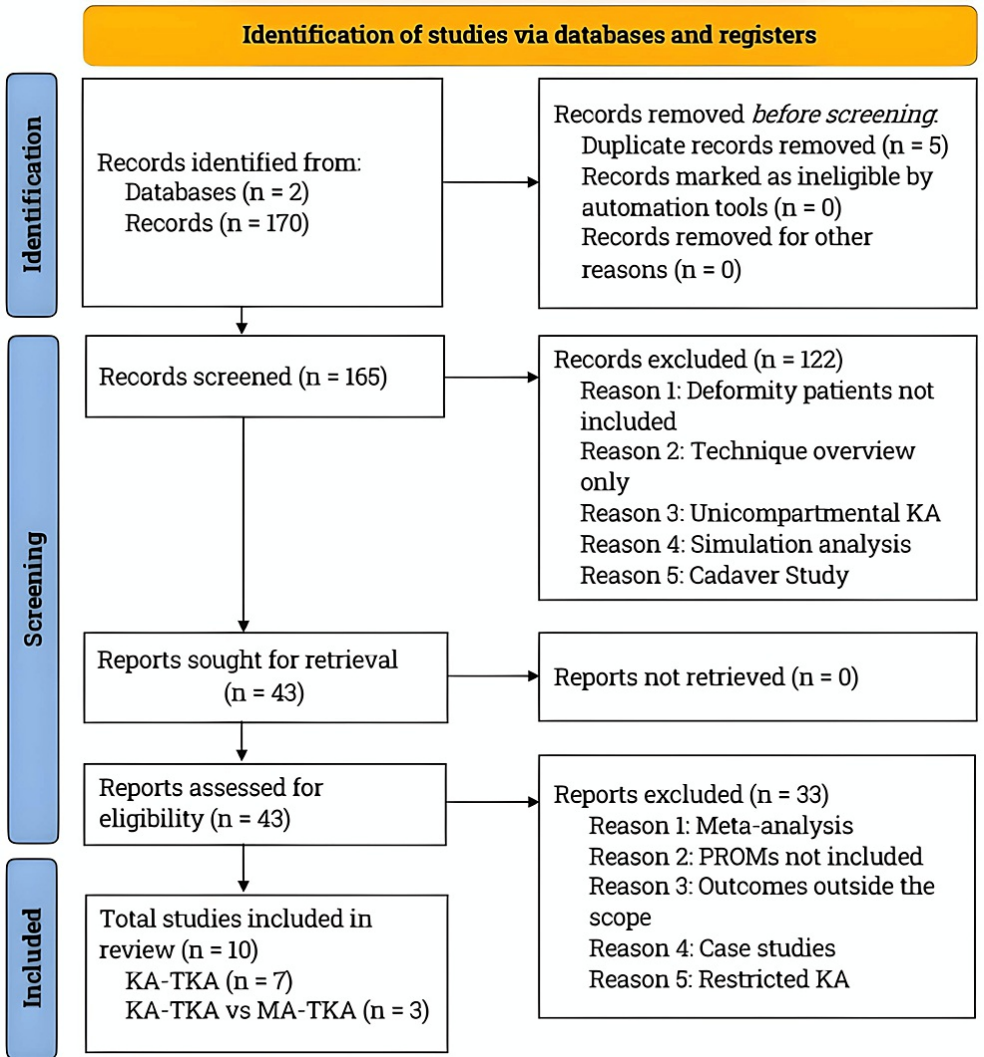
PRISMA flowchart: literature search and study selection process. Following the PRISMA guidelines, we conducted a literature search on PubMed and BioMed Central databases. Our search was focused on studies analyzing the outcomes of KA-TKA in patients with preoperative alignment deformities and those that compared KA-TKA and MA-TKA outcomes in knee osteoarthritis patients. We included relevant studies published in English that met the inclusion criteria of this review paper. KA, kinematic alignment; MA, mechanical alignment; n, number; PRISMA, Preferred Reporting Items for Systematic Reviews and Meta-Analyses; PROMs, patient-reported outcome measures; TKA, total knee arthroplasty

A comprehensive literature search was conducted on PubMed and Biomed Central databases between April and December 2023, in line with the Preferred Reporting Items for Systematic Reviews and Meta-Analyses (PRISMA) guidelines [24]. The study selection process is illustrated in Figure 1.

Clinical evidence levels were assigned to relevant studies published in English, following the previous literature [25].

The inclusion criteria comprised studies analyzing the postoperative TKA outcomes of patients with OA with preoperative varus or valgus deformities who underwent surgeries adhering to the KA philosophy. Studies strictly comparing KA-TKA outcomes of neutral versus varus or valgus alignments and those directly comparing postoperative outcomes of KA-TKA versus MA-TKA were included. Reports of PROMs or implant survivorship were also required. The exclusion criteria included studies employing alignment philosophies other than KA, unicompartmental knee arthroplasty, extra-articular deformity, minimally invasive TKA, and revision TKA. 

Data extraction

Specific keywords were used to extract data. These included “TKA,” “KA,” “varus,” “valgus,” “deformity,” and “outlier.” A complete PubMed search query can be found in Appendices. 

After removing duplicate articles, a title and abstract screening for inclusion criteria was performed. Full-text analysis was then conducted to ensure that all requirements were met. The literature review identified 170 publications in the initial search, of which 43 studies were thoroughly reviewed, and only 10 met all the inclusion criteria. Seven of these studies analyzed KA-TKA outcomes alone, while three directly compared KA-TKA and MA-TKA.

Knee OA

The gradual deterioration of articular cartilage, inflammation, and periarticular and subchondral bone changes characterize knee OA. The pathogenesis of OA is multifactorial, involving mechanical influences, aging, and genetic factors. These risk factors can be broadly categorized into two main mechanisms related to the effects of normal loading on abnormal cartilage and abnormal loading on normal cartilage. These mechanisms cause progressive degeneration and may eventually lead to joint failure [26].

OA affects all structures within the joint, resulting in a loss of articular cartilage, bone remodeling, capsular stretching, and weakening of periarticular muscles. Patients typically experience functional impairment, including stiffness, loss of mobility, and pain and inflammation. Pain associated with OA is usually activity-dependent and can limit daily activities, such as climbing stairs, standing up from a chair, and walking long distances [27].

Diagnosis of knee OA typically involves thoroughly examining the patient for other possible causes of knee pain, including soft tissue tears and chronic inflammation-related disorders. The examination should also include an evaluation of whether the legs are varus (bowlegged) or valgus (knock-kneed), a physical finding that usually indicates malalignment and is highly correlated with disease progression [28]. Radiography is typically indicated for diagnosis and can confirm a loss of articular joint space. However, radiographic findings may not correlate well with the severity of patient-reported pain, and radiographs may appear normal in patients with the disease [29].

Surgical management of knee OA

TKA is the standard surgical intervention for treating severe knee arthritis. TKA has been shown to provide patients with pain relief, functional improvement, and an overall increase in quality of life. Indications for TKA include, but are not limited to, severe knee pain and stiffness that restrict basic activities such as walking, knee pain at rest, chronic swelling, knee deformity, and the failure of conservative treatments such as anti-inflammatory medication, intra-articular injection, physical therapy, or less invasive surgery [30].

The primary goal of TKA is to replace the deteriorated cartilage and bone with an artificial implant that compensates for the wear or damage. The procedure replaces all three knee joint articular components: the distal femur, proximal tibia, and patella. Multiple knee prostheses are available, each with various sizes, component materials, and fixation methods. Typically, biocompatible metal alloys are used for the femoral and tibial components. However, the patellar component and tibial articular insert are comprised of polyethylene to separate the metallic surfaces and provide smooth movements. One significant difference between implant designs is whether the implant requires the removal of the posterior cruciate ligament (PCL). These are designated cruciate retaining (CR) if the PCL is healthy and can be salvaged or posterior stabilizing (PS) if the PCL must be removed and additional stabilization is required in the implant design [31].

The precise placement of implants is paramount in achieving successful TKA. The implants’ alignment depends on seven bony cuts made during the procedure along the femur’s distal, anterior, and posterior margins, the anterior and posterior chamber, across the tibia, and the patella. The accuracy of each cut affects the precision of prosthesis placement and eventual limb alignment, which, in turn, is critical for the procedure’s success [31].

Surgeons have developed several techniques to enhance the accuracy of implant placement and improve patient outcomes. One such method is patient-specific instrumentation (PSI), which employs magnetic resonance imaging (MRI) and computed tomography (CT) to create cutting guides that are customized to the patient’s anatomy [32]. Another popular technique is computer-assisted surgery (CAS), which utilizes intraoperative technology to map patient landmarks and guide appropriate cut orientation. These techniques and traditional manual instrumentation can be used to achieve the desired surgical alignment, whether following an MA or KA philosophy [33].

Leg axis definitions

Comprehending the commonly used lower limb axes is essential to differentiate between the MA and KA philosophies. The femur's mechanical axis (FMA) is defined by a line that runs from the center of the femoral head to the center of the knee joint. The tibia's mechanical axis (TMA) is a line that runs from the center of the knee joint to the center of the ankle joint. The leg's mechanical axis is defined by a line that connects the center of the femoral head to the center of the ankle joint. The hip-knee-ankle (HKA) angle is measured between the FMA and TMA and is then compared to a neutral or 180° mechanical leg axis [34].

The tibiofemoral joint line lies between the distal femoral and proximal tibia bone. The angle formed between the mechanical axis of the femur and this joint line is referred to as the lateral distal femoral angle (LDFA). Similarly, the angle between the mechanical axis of the tibia and this joint line is known as the medial proximal tibial angle (MPTA). On an average person, these two angles are not perpendicular to their corresponding bone mechanical axes. The LDFA typically exhibits a 3° valgus orientation compared to the FMA, whereas the MPTA is generally angled at 3° varus compared to the TMA [34]. It is important to note that these angles can vary from one individual to another [35].

MA overview

The ultimate objective of an MA-TKA procedure is to achieve proper leg alignment with a neutral mechanical axis and an HKA angle of 180°. This is accomplished by making perpendicular cuts to the distal femoral and proximal tibial bones along their respective mechanical axes, resulting in an LDFA and MPTA of 90°. Although the primary aim is for these angles to be perpendicular to the mechanical axis, deviations of up to ±3° from a neutral mechanical axis are generally acceptable. An HKA angle of less than 177°, measured medially from a 180° axis, is indicative of varus alignment, while an HKA angle of less than 177°, measured laterally from a 180° axis, is indicative of valgus alignment. Achieving neutral alignment is critical in ensuring even weight distribution, proper loading, and long-term implant survival [4].

Various surgical techniques are employed to perform an MA-TKA. These techniques share a common objective of achieving neutral mechanical alignment. However, they differ in the sequence of bone cuts executed and how the rotation of the implant and knee ligament balancing is attained. The tibiofemoral joint line is not usually perpendicular to the mechanical axis, so any alterations made to the LDFA and MPTA to achieve a neutral MA may affect the soft tissue tension in the knee. Therefore, ligament balancing strategies are implemented to achieve even tension on the medial and lateral sides during flexion and extension to ensure joint stability. Maintaining consistent medial and lateral ligament tension is crucial for successful MA-TKA outcomes [36].

KA overview

A KA-TKA procedure aims to restore the patient’s native joint kinematics, which were present before the onset of arthritis. This procedure preserves the patient’s femorotibial joint line, leaving the LDFA, MPTA, and HKA unaltered. Since these angles remain unchanged, ligament releases are often unnecessary, and the native knee’s medial and lateral tibial compartment forces are restored [37].

During a KA-TKA procedure, ensuring that the resected bone and cartilage’s thickness is equal to the implant’s thickness at the distal and posterior cuts of the femoral condyles and each tibial plateau is imperative. In cases where preoperative varus or valgus deformity exists, the patient should be left with the same degree of deformity postoperatively to maintain the patient’s individual femorotibial joint line. Therefore, KA-TKA aims to attain an HKA axis that matches the patient’s preoperative alignment rather than achieving an HKA angle of 180°. Proponents of the KA philosophy argue that it provides a more natural or physiologic prosthetic knee function and ultimately improves patient satisfaction [37].

PROMs overview

PROMs are frequently employed to assess the efficacy of surgical procedures concerning patient function and satisfaction. In particular, researchers investigating the effectiveness of MA-TKA and KA-TKA utilize the following PROMs:

The Knee Injury and Osteoarthritis Outcome Score (KOOS) is self-administered. It assesses five outcomes: pain, symptoms, activities of daily living, sport and recreation function, and knee-related quality of life [38].

The Oxford-12 Item Knee Score (OKS) is a PROM developed for knee arthroplasty procedures. Its primary aim is to evaluate an individual’s postoperative functional capacity and pain levels. The OKS questionnaire comprises 12 items that focus on assessing the patient’s ability to perform activities of daily living and the severity of pain experienced during these activities [39].

The Western Ontario and McMaster Universities Arthritis Index (WOMAC) is a disease-specific self-report questionnaire assessing pain, stiffness, and physical disability in patients with OA of either the hip or the knee [40].

The visual analog scale (VAS) of pain intensity consists of a line with two descriptors representing extremes of pain intensity at each end. Patients rate their pain intensity by making a mark on the line, and the VAS is scored by measuring the distance from the end of the line. VASs are among the most used measures of pain intensity in clinical trials [41].

The Veterans Rand 12-item Survey (VR-12) is a self-reported outcome measure assessing the impact of health on an individual’s everyday life. It is often used as a quality-of-life measure. Questions relate to limitations on daily activities, pain, fatigue, and general mental health [42].

The Forgotten Joint Score (FJS) is a method utilized to evaluate a patient’s ability to forget about their artificial joint in their daily routine. The questionnaire concentrates on the patient’s awareness of a particular joint, considering intense sensations such as pain and more subtle feelings like mild stiffness, subjective dysfunction, or discomfort [43].

The Knee Society Score (KSS) is a widely used outcome measure that combines an objective physician-derived component with a patient-derived subjective element. The score evaluates pain relief, functional abilities, satisfaction, and fulfillment of expectations. The objective clinical aspects of the score are represented by the Knee Society C (KS-C), while the subjective functional elements are represented by the Knee Society F (KS-F). In some cases, the KS-C and KS-F components of the KSS are reported separately, while in others, they are combined to form the KSSC [44].

The Mean Clinical Important Difference (MCID) is a concept that represents the minimum level of improvement that is deemed significant by a patient. This notion has been put forth as a new standard for evaluating the efficacy of a given treatment and describing the level of patient satisfaction with said treatment. By using the MCID as a benchmark, healthcare providers can better assess the benefits of a treatment and make informed decisions in the best interest of their patients [45].

Clinical evidence of KA-TKA efficacy in patients with knee OA

As the pioneer of the KA-TKA technique, Howell et al. published several studies over the last decade analyzing the clinical and functional outcomes of his KA-TKA patients. These studies aimed to determine whether function and implant survivorship differed when the tibial component, knee, and limb alignment were in a specified normal range, varus outlier, or valgus outlier postoperatively [46-50].

Alignment categories were established from a long-leg scanogram. Definitions include the component as in range (less than and equal to 0°) or a varus outlier (>0°), alignment of the knee as in range (between -2.5° and -7.4° valgus) or a varus (>-2.5°) or valgus (<-7.4°) outlier, and alignment of the limb as in range (0° ± 3°) or a varus (>3°) or valgus (<-3°) outlier. Function was assessed using the OKS and WOMAC scores, and catastrophic failure was reported as the incidence of revision attributable to loosening, wear, and instability of the femoral or tibial components [46-50].

Howell et al. conducted a prospective study to investigate the efficacy of KA-TKA using PSI guides. The study followed 198 patients with 214 knees who underwent TKAs using a CR prosthesis between February 2008 and October 2008. The minimum postoperative follow-up period was 31 months, ranging from 31 to 43 months. The study found that the mean OKS and WOMAC scores for TKAs grouped in the outlier categories were either the same or slightly higher than those in the in-range categories for tibial component, knee, and limb alignment, although the differences were insignificant. The overall mean OKS improved by 23 points from a preoperative score of 20 to a 31-month follow-up score of 43. The overall mean WOMAC score was 92 (95% confidence interval [CI] 90-94). The incidence of catastrophic failure was zero for the in-range and outlier categories of the tibial component, knee, and limb alignment. There were three reoperations (1.4%) across all categories, and no patient reported revision surgery for femoral or tibial component loosening, wear, or instability [46].

The study concluded that KA-TKA is an effective method for restoring function without catastrophic failure, regardless of the established alignment category. The data showing high function and limited incidence of catastrophic failure in the varus outlier category can be used to counter the concern that KA may compromise function and place the components at high risk for catastrophic failure [46].

Howell et al. conducted another prospective study evaluating the efficacy of KA-TKA performed using generic instruments. The study comprised 101 patients (101 knees) who underwent the procedure between June 2012 and September 2012 and were monitored for at least six months, ranging from 6 to 8 [47].

The study revealed that the patients exhibited a mean OKS of 42 ± 5.2 and a WOMAC score of 89 ± 11.2 following the procedure. The functional outcome for patients categorized as an outlier or in-range for each alignment category was the same. The results indicated no significant difference in OKS between in-range and outlier groups classified using parameters of limb alignment (42 ± 5 versus 44 ± 3) and tibial component alignment (44 ± 3 versus 42 ± 5). Additionally, the study found no significant difference in WOMAC scores between in-range and outlier groups categorized using parameters of limb alignment (89±11 versus 95±5) and tibial component alignment (95 ± 3 versus 89 ± 11) [47].

The study concluded that using generic instruments for KA-TKA results in high functional restoration, regardless of whether patients had an alignment categorized as an outlier or in-range. However, it must be noted that the study did not assess the extended-term endurance of the implant and the patient’s continued functionality in the long run [47].

A few years later, Howell et al. published a prospective study with a relatively extensive sample size to evaluate varus alignment’s impact on knee arthroplasty procedures’ performance. The study followed 214 consecutive patients (219 knees) who underwent KA-TKA in 2007 with a mean postoperative follow-up of 6.3 years, ranging from 5.8 to 7.2 years [48].

The study revealed that varus alignment of the tibial component, knee, and limb did not significantly affect the implant’s survival or function. The average OKS was 43, and the average WOMAC score was 91. The function of tibial components, knees, and limbs aligned in varus was comparable to patients aligned in-range. The mean OKS and WOMAC scores did not differ between patients grouped as varus, valgus, or in-range according to the tibial component, knee, and limb alignment. The implant survivorship was 97.5%. Three implants had been revised due to deep infection, a loose tibial component, and patella instability. Two loose patella components were pending revision and considered failures [48].

The study’s findings suggest that KA can be considered an alternative to MA for performing primary TKA. The results of this study support the notion that varus alignment of the tibial component, knee, and limb does not adversely affect implant survival and function at a mean of 6.3 years after KA-TKA [48].

Howell et al. (2018) conducted a retrospective analysis of 217 patients (222 knees) who underwent primary KA-TKA with PSI in 2007 and were followed up for 10 years. The study aimed to evaluate the impact of postoperative alignment of the tibial component, knee, and limb on implant survival, revision rate, and level of function. The patients had no restrictions on preoperative deformity, and the goal of KA-TKA was to restore native joint lines and limb alignment [49].

The study revealed a high implant survivorship rate of 97.4% for revision for any reason and 98.4% for aseptic failure. The postoperative alignment of the tibial component, knee, and limb did not affect implant survival (*P* = 0.2288-0.4164). Patients classified as varus outliers, valgus outliers, and in-range had similar implant survival and function scores. The OKS averaged 43 (95% CI 42.4-44.4), and the WOMAC score averaged 7 (95% CI 5.5-9.3). The postoperative alignment of the tibial component, knee, and limb did not affect the mean OKS and WOMAC scores (*P* = 0.0530-0.3596) [49].

Therefore, the study concluded that KA-TKA with PSI, without restrictions on the preoperative deformity and postoperative alignment of the tibial component, knee, and limb in the varus and valgus outlier ranges did not adversely affect the ten-year implant survival, yearly revision rate, and level of function [49].

Howell et al. conducted a comprehensive study to assess the outcomes of primary TKA using an unrestricted KA technique. The study involved 1,117 consecutive patients who underwent KA-TKA between September 2014 and September 2017. The researchers employed a different methodology to categorize the postoperative outcomes compared to previous studies. The FMA, TMA, and HKA were measured in both legs, and each alignment angle was assigned to one of five FMA, five TMA, and seven HKA phenotype categories [50].

The study found that the median FJS was similar across all phenotype categories. The median OKS was identical between the TMA and HKA phenotypes and highest in the most varus FMA phenotype. The FJS did not show significant differences among the FMA, TMA, and HKA phenotypes (*P* = 0.586, 0.971, and 0.858, respectively). Similarly, the OKS did not exhibit significant differences between the TMA and HKA phenotypes (*P* = 0.221 and 0.295, respectively). Notably, the most varus FMA phenotype was associated with a greater OKS than three other FMA phenotypes (*P* = 0.029). Three TKAs (1.5%) required reoperation for anterior knee pain or patellofemoral instability in the subgroup of patients with more valgus phenotypes. However, the researchers found no implant revisions for component loosening, wear, or tibiofemoral instability [50].

The study concluded that using the unrestricted KA technique (known as the calipered technique), to restore a wide range of phenotypes, resulted in satisfactory FJS and OKS scores without implant revision at a mean follow-up of four years. Each phenotype showed a comparable and high median FJS and OKS. However, the three reoperated patients had a more valgus setting of the prosthetic trochlea than recommended for mechanical alignment. The researchers suggested that designing a femoral component specifically for KA that restores patellofemoral kinematics with all phenotypes, particularly the more valgus ones, is a viable strategy to reduce the risk of reoperation [50].

Notably, the function and failure rates presented by designing surgeons in published papers tend to report superior outcomes than those reported by registries [51]. Therefore, it is imperative to conduct independent validation and continued research to establish the outcomes reported by Howell et al. (2021) [50]. In addition, it should be noted that the alignment categories in all five studies were established based on postoperative knee angles [46-50]. Thus, it is unclear whether the preoperative varus or valgus alignment of the limb would follow the same pattern of results as the postoperative categories established in the studies. Further research is necessary to clarify this aspect.

Hsu et al. evaluated clinical and functional outcomes in patients who underwent KA-TKA using generic instrumentation between September 2014 and September 2017. The study involved 123 participants who underwent 140 KA-TKAs and were categorized based on five common knee phenotypes for KA-TKA alignment target setting. All TKAs were performed with alignment targets set according to the original phenotypes of the knee. The researchers then collected and analyzed patients’ one-year range of motion (ROM), OKS, and KSSC [52].

The study findings indicated that the three-year survivorship rate was 99.3% for all causes of revision and 100% survivorship with revision other than infection as the endpoint. No aseptic loosening or instability was observed. Although there was a significant difference in postoperative alignment parameters between the five knee phenotypes (p < 0.05), similar functional improvement was observed in all patients at the one-year follow-up. The study also found no significant difference in functional outcomes, including OKS (*P* = 0.570), KSSC (*P* = 0.809), and ROM (*P* = 0.529), between the five knee phenotypes [52].

The study results support individualizing alignment targets based on the original (native) knee phenotype. Regardless of the postoperative alignment category, patients reported similar functional improvement. Therefore, the findings suggest that individualized alignment targets can be adopted to achieve the desired outcomes in patients undergoing KA-TKA [52].

Bar-Ziv et al. conducted a retrospective, single-center study to evaluate the outcomes of KA-TKA in patients with preoperative valgus knee deformities. The study was conducted between January 2018 and March 2020, and the patient cohort was categorized into two groups based on preoperative limb alignment: valgus and non-valgus groups. The researchers analyzed patient-reported outcomes at least two years following KA-TKAs for valgus knees (*n* = 51) and compared the results to KA-TKA performed for non-valgus knees (*n* = 275). The VAS, OKS, and KOOS outcome measures were used, and MCID was used for OKS and KOOS [53].

The study results indicated that the patients in both groups demonstrated significant improvements in pain relief and functionality three years after the surgical procedure. However, the average scores were superior in the non-valgus group for VAS (*P* = 0.005), OKS (*P* = 0.013), and KOOS (*P* = 0.009). Nevertheless, these differences did not translate to statistically significant differences in MCID achievement rates. The revision rates between the two groups were not significantly different (*P* = 1.00). Only two cases in the non-valgus group required revision for periprosthetic joint infection and patellar dislocation (0.7%). In contrast, no revision cases were in the valgus group (0%) [53].

The study findings showcased that KA-TKA efficiently relieved pain and restored function in patients with valgus alignment. While patients with a preoperative valgus alignment had statistically significant inferior patient-reported outcome scores and inferior improvement scores compared to patients without valgus alignment, these differences did not reach statistically significant differences in MCID achievement rates [53].

It is noteworthy that the follow-up period in this study was relatively short and may not provide a comprehensive assessment of the long-term outcomes of KA-TKA in patients with valgus knee OA. Furthermore, due to the sample size, it was not feasible to subcategorize the valgus group to compare valgus deformity phenotypes. These limitations require further investigation in future studies [53].

Table 1 highlights the critical aspects of the reviewed studies that demonstrate the efficacy of KA-TKA in treating patients with knee OA.

**Table 1:** Effectiveness of KA-TKA in treating patients with knee OA. The table provides an overview of the key aspects extracted from various studies that explore the effectiveness of KA-TKA in treating patients with knee OA. CR, cruciate retaining; FJS, Forgotten Joint Score; KA-TKA, kinematic alignment-total knee arthroplasty; KOOS, Knee Injury and Osteoarthritis Outcome Score Joint Replacement; KSS-C, Knee Society Score Clinical Score; MCID, mean clinical important difference; OA, osteoarthritis; OKS, Oxford Knee Score; PSI, patient-specific instrumentation; ROM, range of motion; VAS, visual analog scale; WOMAC, Western Ontario and McMaster Universities Arthritis Index

Author	Level of clinical evidence	Sample size	KA method	Follow-up time	Assessment tool	Findings
Howell et al. [46]	IV	198 (214 knees)	PSI CR	38 months (31-43)	OKS	No significant difference was found in OKS between in-range, varus outlier, or valgus outlier groups categorized using parameters of tibial component alignment (*P* = 0.4491), knee alignment (*P* = 0.1150), and limb alignment (*P* = 0.2316).
					WOMAC	No significant difference was found in OKS among the groups categorized by tibial, knee, and limb component alignment parameters, including in-range, varus outlier, and valgus outlier groups (*P* = 0.6035, *P* = 0.1602, and *P* = 0.2320, respectively).
					Implant Survivorship	No catastrophic failure cases were recorded, and the survivorship rate for all reasons (excluding deep infection) stood at 98.6%.
Howell et al. [47]	IV	101 (101 knees)	Calipered CR	Six years (6-8)	OKS	No significant difference in OKS was observed between the in-range and outlier groups, classified based on parameters of limb and tibial component alignment (42 ± 5 versus 44 ± 3 and 44 ± 3 versus 42 ± 5, respectively).
					WOMAC	No significant difference in WOMAC was found between the in-range and outlier groups, categorized based on limb alignment (89 ± 11 versus 95 ± 5) and tibial component alignment (95 ± 3 versus 89 ± 11).
					Implant Survivorship	No catastrophic failure cases were recorded, and the implant survivorship was 100%.
Howell et al. [48]	III	214 (219 knees)	PSI CR	6.3 years (5.8-7.2)	OKS	No statistically significant difference was found in the OKS among groups categorized using parameters of tibial component alignment (*P* = 0.6658), knee alignment (*P* = 0.8261), and limb alignment (*P* = 0.9379), including in-range, varus outlier, and valgus outlier groups.
					WOMAC	No significant difference was found in OKS between the in-range, varus outlier, or valgus outlier groups that were categorized using the parameters of tibial component alignment (*P* = 0.9012), knee alignment (*P* = 0.8721), and limb alignment (*P* = 0.7945).
					Implant Survivorship	Implant survivorship was reported as 97.5% for revision for any reason.
Howell et al. [49]	III	216 (220 knees)	PSI CR	10 years	OKS	No significant difference was observed in the OKS between the in-range, varus outlier, or valgus outlier groups when categorized using the parameters of tibial component alignment (*P* = 0.3494), knee alignment (*P* = 0.0917), and limb alignment (*P* = 0.1073).
					WOMAC	No significant difference in OKS was observed between the in-range, varus outlier, or valgus outlier groups, categorized based on the parameters of tibial component alignment (*P* = 0.3596), knee alignment (*P* = 0.0824), and limb alignment (*P* = 0.0530).
					Implant Survivorship	Implant survivorship was 97.4% for revision for any reason and 98.4% for aseptic failure.
Howell et al. [50]	III	198 (198 knees)	Calipered CR	Four years	FJS	FJS showed no statistical differences between the FMA, TMA, and HKA phenotypes (*P* = 0.586, 0.971, and 0.858, respectively).
					OKS	OKS was not significantly different between the TMA and HKA phenotypes (*P* = 0.221 and 0.295, respectively). The most varus FMA phenotype was associated with a greater OKS than three other FMA phenotypes (*P* = 0.029).
					Implant Survivorship	The implant survivorship across all categories was 95.5%.
Hsu et al. [52]	III	123 (140 knees)	Calipered PS	36.5 months (21-42)	OKS	There was no significant difference in OKS across all five phenotypes (*P* = 0.570).
					KSS-C	There was no noticeable distinction in KSS-C among the five phenotypes, as indicated by the statistical analysis (*P* = 0.809).
					ROM	No significant difference in ROM was observed among all five phenotypes (*P* = 0.529).
					Implant Survivorship	Implant survivorship was reported as 99.3% for all causes of revision and 100% with revision other than infection.
Bar-Ziv et al. [53]	III	326 (326 knees)	Calipered PS	3.34 ± 0.79 years (non-valgus) and 3.17 ± 0.83 years (valgus)	VAS	The VAS scores significantly differed between the non-valgus and valgus groups (*P* = 0.005).
					OKS	OKS significantly differed in the non-valgus versus valgus group (p = 0.013).
					KOOS	KOOS symptoms score showed a significant difference between the non-valgus versus valgus group (*P* = 0.009); KOOS pain score showed a significant difference in the non-valgus versus valgus group (*P* = 0.042); KOOS function score showed a considerable difference between the non-valgus versus valgus group (*P* = 0.041); KOOS QOL score showed a significant difference between the non-valgus versus valgus group (*P* = 0.017); KOOS total score showed no significant difference between the non-valgus versus valgus group (*P* = 0.072).
					MCID	No significant difference in MCID achievement rates was found among OKS (*P* = 0.707), KOOS symptoms (*P* = 0.831), KOOS pain (*P* = 0.189), KOOS function (*P* = 0.654), and KOOS QOL (*P* = 0.804).
					Implant Survivorship	Survivorship for the implant was 99.3% in the non-valgus group and 100% in the valgus group, with no significant differences in revision rates (*P* = 1.00).

Efficacy of MA-TKA versus KA-TKA in patients with knee OA

Luan et al. conducted a study to determine the patient characteristics that would make a KA-TKA procedure more suitable than an MA-TKA procedure. The study involved a review of 296 consecutive patients who received unilateral TKA using a computer-assisted navigation system from 2016 to 2018. The study participants were divided into two groups: 114 underwent KA-TKA, while 182 underwent MA-TKA. The clinical outcomes of the two groups were then compared using a ROM and KSS [54].

The ROM (*P* = 0.752) and KSS (*P* = 0.0107) scores showed no significant difference between the KA-TKA and MA-TKA groups at the one-year follow-up. However, the analysis revealed that obesity and preoperative HKA angle were both factors that influenced the association between alignment techniques and total KSS score. Patients with a body mass index (BMI) of over 30 kg/m^2^ who underwent MA-TKA achieved better KSS scores at the one-year postoperative mark than patients with the same BMI who underwent KA-TKA (*P* < 0.05). Conversely, patients with preoperative HKA angle greater than 10° varus achieved better KSS scores with KA-TKA than MA-TKA (*P* < 0.05) [54].

The study concluded that obese patients may benefit more from MA-TKA, while patients with severe varus deformity may have better outcomes with KA-TKA. The study also found that all other patient characteristics analyzed had comparable outcomes for KA-TKA and MA-TKA. It is worth noting that the KA-TKA technique used in the study was limited to intraoperative angles of 6° varus and 3° valgus. Therefore, these findings may only apply to KA-TKA procedures with a broader alignment cutoff or an unrestricted KA approach [54].

Elbuluk et al. conducted a study to compare PROMs in patients with varus OA who underwent TKA with either an MA target or a KA target. The study utilized the same implant and technological guidance for both groups. One hundred consecutive patients who underwent MA-TKA were matched 1:1 for age, gender, BMI, and varus deformity, with 100 patients who underwent KA-TKA between 2016 and 2019. Patient-reported outcomes were assessed using a VAS, VR-12, KOOS ROM, and FJS. The scores were measured preoperatively, at six weeks, 12 months, and 24 months postoperatively [55].

The study results showed that patients who underwent KA-TKA had significantly higher FJS scores than those who underwent MA-TKA at one and two years postoperatively (*P* < 0.001). At six months postoperatively, the FJS scores were statistically similar between the two groups (*P* = 0.5). The VAS scores were significantly lower in the KA group at six weeks postoperatively compared to the MA group (*P* = 0.04). However, there was no statistical difference in VAS scores between the two groups at one-year (*P* = 0.05) and two-year (*P* = 0.05) follow-ups. The KOOS Junior score was significantly higher in the KA group at six weeks (*P* = 0.05), one year (*P* = 0.05), and two years (*P* = 0.05). The two groups had no statistically significant difference in knee ROM (*P* = 0.55). The six-week VR-12 scores were also statistically similar between the two groups (*P* = 0.32) [55].

The study results suggested that KA-TKA patients experienced less pain in the first six weeks after surgery, as demonstrated by lower VAS scores, and improved function at one and two years, as indicated by higher FJS scores. Other outcome measures at the study time points were comparable between the matched MA-TKA and KA-TKA patients. However, it is vital to note that the study focused solely on varus knee phenotypes, and therefore, the findings may not be translatable to preoperative valgus knee phenotypes. Furthermore, the study did not analyze the degree of varus deformity, making it challenging to conclude whether the results can be translated to all preoperative varus phenotypes [55].

Wen et al. conducted a comparative study to evaluate the early clinical outcomes of KA-TKA with manual instrumentation and MA-TKA in a patient population from China. A retrospective analysis was performed on 126 patients who underwent unilateral TKAs for knee OA with a varus deformity from October 2018 to September 2020. Knee Society Score Clinical Score (KSS-C), Knee Society Score Functional Score (KSS-F), and FJS were measured and statistically analyzed three months and two years after surgery [56].

The study enrolled 65 patients who underwent KA-TKA and 61 who underwent MA-TKA. At three months after surgery, the KA-TKA group achieved significantly higher scores than the MA-TKA group for KSS-C (79.01 ± 6.44 versus 74.03 ± 6.18), KSS-F (74.61 ± 9.50 versus 70.82 ± 8.96), and FJS (74.20 ± 7.38 versus 67.36 ± 9.82), *P* < 0.05, respectively. At the two-year follow-up, the KA-TKA group continued to achieve significantly higher scores than the MA-TKA group for KSS-C (84.89 ± 8.59 versus 78.37 ± 9.32) and FJS (86.43 ± 9.65 versus 73.12 ± 13.82), *P* < 0.05, respectively. However, there was no statistical difference between KSS-F scores (81.15 ± 9.51 versus 78.03 ± 10.62), *P* = 0.081 [56].

The study results suggest that KA-TKA is superior to MA-TKA in terms of clinical performance, knee function, and subjective sensation at three months and up to two years after surgery. However, it is worth noting that the study only included patients with preoperative varus knees, and therefore, the results may not apply to patients with valgus knee deformities. Moreover, the degree of varus deformity was not analyzed in detail, limiting the study findings generalizability. Further research is needed to confirm these results and to explore the potential benefits of KA-TKA in a broader patient population [56].

Table 2 highlights the critical aspects of the reviewed studies that compare the efficacy of KA-TKA with MA-TKA in treating patients with knee OA.

**Table 2:** KA-TKA versus MA-TKA in treating patients with varus OA. The table highlights the studies directly comparing the efficacy of KA-TKA and MA-TKA in treating patients with varus OA. FJS, Forgotten Joint Score; KA-TKA, kinematic alignment-total knee arthroplasty; KOOS JR, Knee Injury and Osteoarthritis Outcome Score Joint Replacement Junior; KSS, Knee Society Score; KSS-C, Knee Society Score Clinical Score; KSS-F, Knee Society Score Functional Score; MA-TKA, Mechanical Alignment-Total Knee Arthroplasty; OA, osteoarthritis; ROM, range of motion; VAS, visual analog scale; VR-12, Veterans Rand 12-item Survey

Author	Level of Clinical Evidence	Sample Size	Follow-up time	Assessment Tool	Findings
Luan et al. [54]	II	114 patients (KA-TKA) and 182 patients (MA-TKA)	12 months	KSS	KSS did not statistically differ in patients who underwent KA-TKA and MA-TKA (*P* = 0.107).
				ROM	ROM did not statistically differ in patients who underwent KA-TKA and MA-TKA (*P* = 0.752).
Elbuluk et al. [55]	II	100 patients (KA-TKA) and 100 patients (MA-TKA)	6 weeks, 12 months, and 24 months	FJS	The average FJS was not statistically different at six weeks (*P* = 0.5). However, the average FJS was significantly higher in patients who underwent KA-TKA than MA-TKA at 12 (*P* < 0.001) and 24 months (*P* < 0.001).
				VAS	The patients who underwent KA-TKA had significantly lower VAS scores than those who underwent MA-TKA at six weeks (*P* = 9.04). However, there was no significant difference at 12 and 24 months (*P* = 0.5).
				KOOS JR	The average KOOS JR score was significantly higher for patients who underwent KA-TKA than those who received MA-TKA at six weeks (*P* = 0.05), 12 (*P* = 0.05), and 24 months (*P* = 0.05).
				ROM	The ROM did not have a significant difference at 6 weeks (*P* = 0.55), 12 (*P* = 0.55), and 24 months (*P* = 0.55).
				VR-12	There was no significant difference in patients’ VR-12 scores at six weeks (*P* = 0.32).
Wen et al. [56]	III	65 patients (KA-TKA) and 61 patients (MA-TKA)	Three months and 24 months	KSS-C	The three-month and 24-month follow-ups showed that patients who underwent KA-TKA had significantly higher average KSS-C scores than those who underwent MA-TKA, with *P* < 0.05 for both.
				KSS-F	The KSS-F score was significantly higher in patients who underwent KA-TKA than those who received MA-TKA at the three-month and 24-month follow-ups (*P* < 0.05).
				FJS	The average FJS was not significantly different at the three-month and 24-month follow-ups, *P* = 0.081, respectively.

Discussion

The critical finding of the studies that directly compared KA and MA surgical outcomes for patients with preoperative deformities was that patients who received KA-TKA had significantly better functional outcomes than those who underwent MA-TKA. Elbuluk et al. showed significantly better FJS at 12 and 24 months, VAS at six weeks, and KOOS Junior at six weeks and 12 and 24 months [55]. Wen et al. showed significantly better KSS-C, KSS-F, and FJS at three and 24 months [56]. Luan et al. also showed patients with an HKA angle of more than 10° varus benefited more from KA than MA about one-year KSS measures [54].

The review of the literature analyzing postoperative outcomes of KA-TKA patients with preoperative varus or valgus deformities demonstrated that the overall postoperative alignment had little clinical significance in determining functional outcomes and survivorship. In several studies, the Howell research group established that KA-TKA, with PSI or manual instrumentation, restores high function with limited failure, regardless of postoperative outlier alignment. These results were shown at six months, three years, four years, six years, and 10 years postoperatively [46-50].

Hsu et al. also showed comparable functional improvement and survivorship with KA-TKA regardless of original knee phenotype and varied postoperative alignment [52]. Bar-Ziv et al. analyzed preoperative valgus KA-TKA patients. While this cohort may have significantly inferior PROMs compared with non-valgus patients, these differences did not reach statistically significant differences in MCID achievement rates. In addition, regardless of preoperative alignment, considerable improvement in pain and function was seen in both groups, with 100% implant survivorship at three-year follow-ups in the valgus group [53].

Utilizing individualized KA alignment strategies may have an advantage over patients who undergo MA-TKA. Data showing appropriate survivorship of KA-TKA, independent of deformity severity, should encourage surgeons to incorporate these alignment strategies in their surgical practice to improve their patient’s functional outcomes. Additional long-term data collected in the future will strengthen the evidence and may also influence the design of future implants to better support a KA-TKA alignment.

Surgeons should consider incorporating these strategies to optimize implant survivorship and functional recovery, regardless of the severity of preoperative deformity. Additionally, the data presented in this literature review may influence future implant design to better support KA-TKA alignment. Further long-term studies are needed to strengthen the evidence for KA-TKA and to better understand the optimal alignment strategies for individual patients. Nonetheless, the findings of this literature review suggest that KA-TKA may be a viable alternative to MA-TKA for patients with preoperative deformities, and it may provide comparable, if not superior, functional outcomes and implant survivorship.

Limitations

During the literature search, several limitations were encountered. It was noted that limited studies outline the outcomes of preoperative deformity patients with an MA-TKA control compared to the KA-TKA outcomes. Only three studies matched this criterion, and these studies also analyzed varus phenotypes. Therefore, the findings of these studies may not be generalizable to preoperative valgus knee phenotypes. While statistically significant differences were shown in several categories, it was unclear whether these differences translated into substantial differences in MCID achievement rates. The severity of the varus deformity was not categorized across several reviewed studies, making it difficult to conclude whether these results can be translated to all preoperative varus phenotypes.

Of the 10 papers reviewed, seven studies only analyzed KA-TKA patients, categorizing them based on their pre- or postoperative alignment. These studies did not include a control group of MA-TKA patients, thus not allowing the evaluation of the impact of the reported success of the KA-TKA compared to MA-TKA. It is worth noting that the same group of researchers published five of these studies. As previously mentioned, alignment and functional outcomes reported by designing surgeons are generally better than those reported by registries.

Overall, achieving homogeneity in the study design was challenging in this set of studies evaluating MA-TKA and KA-TKA in patients with knee OA. Many of these studies had small sample sizes for individual alignment categories. Seven out of 10 papers should have included reported outcomes in the past three and a half years. However, the lack of long-term follow-up means that incidences of failure and patient functions may change.

## Conclusions

The available clinical evidence suggests that KA-TKA is an effective treatment option for patients with knee OA. Phenotypical alignment, which refers to a person's natural knee alignment, has little clinical significance concerning functional outcomes and implant survivorship after KA-TKA. In addition, surgical outcomes for patients with a preoperative deformity who underwent KA-TKA were comparable to those who underwent MA-TKA. In certain aspects, KA-TKA yielded significantly better functional outcomes than MA-TKA. Nevertheless, studies with extensive sample sizes and long-term follow-ups directly comparing KA-TKA with MA-TKA in treating patients with knee OA are necessary to comprehensively demonstrate the effectiveness of one technique over the other. Moreover, further investigation into the impact of KA-TKA on implant survivorship will provide a better understanding of the technique's benefits and ultimately lead to better patient outcomes.

## Appendices

Please find below the complete search query for the PubMed database intended to identify publications that have analyzed the outcomes of postoperative TKA in patients suffering from knee OA with preoperative varus or valgus deformities. Specifically, we searched for studies that followed a KA philosophy during the surgical procedures and compared KA-TKA with MA-TKA.

(("Arthroplasty, Replacement, Knee"[Mesh] OR "total knee arthroplasty*"[tw] OR "knee arthroplasty*"[tw] OR "knee replacement*"[tw] OR "total knee replacement*"[tw] OR "total knee*"[tw] OR "total knee surger*"[tw] OR "TKA"[tw] OR "TKR"[tw]) AND ("kinematic alignment"[tw] OR "kinematic"[tw] OR "alignment philosoph*"[tw])) AND ("varus knee"[tw] OR "valgus knee"[tw] OR "varus deformit*"[tw] OR "valgus deformit*"[tw] OR "knee deformit*"[tw] OR "varus align*"[tw] OR "valgus align*"[tw] OR "constitutional varus"[tw] OR "constitutional valgus"[tw] OR "varus osteoarthritis"[tw] OR "valgus osteoarthritis"[tw] OR "preoperative varus"[tw] OR "preoperative valgus"[tw] OR "pre-operative varus"[tw] OR "pre-operative valgus"[tw] OR "residual varus"[tw] OR "residual valgus"[tw] OR "varus outlier"[tw] OR "valgus outlier"[tw])
